# Invariant natural killer T-cell neutralization is a possible novel therapy for human eosinophilic esophagitis

**DOI:** 10.1038/cti.2013.13

**Published:** 2014-01-10

**Authors:** Madhavi Rayapudi, Priya Rajavelu, Xiang Zhu, Ajay Kaul, Rituraj Niranjan, Scott Dynda, Akanksha Mishra, Jochen Mattner, Asifa Zaidi, Parmesh Dutt, Anil Mishra

**Affiliations:** 1 Department of Pathology, University of Cincinnati, Cincinnati, OH, USA; 2 Division of Pulmonary Biology, Cincinnati Children's Hospital Medical Center, Cincinnati, OH, USA; 3 Division of Allergy and Immunology, Cincinnati Children's Hospital Medical Center, Cincinnati, OH, USA; 4 Division of Gastroenterology, Hepatology, and Nutrition, Cincinnati Children's Hospital Medical Center, Cincinnati, OH, USA; 5 Division of Gastroenterology and Liver Disease, Case Western Reserve University, Cleveland, OH, USA; 6 Biomedical Engineering, Case Western Reserve University, Cleveland, OH, USA; 7 Division of Immunobiology, Cincinnati Children's Hospital Medical Center; Cincinnati, OH, USA; 8 Mikrobiologisches Institut-Klinische Mikrobiologie, Immunologie und Hygiene, Universitätsklinikum Erlangen and Friedrich-Alexander Universität Erlangen-Nürnberg, D-91054, Erlangen, Germany; 9 Pulmonary Diseases, Tulane Eosinophilic Disorder Center, Tulane University School of Medicine, New Orleans, LA, USA

**Keywords:** anti-CD1d, anti-Vα24Jα18, CXCL16, iNKT cells, PBS57

## Abstract

Eosinophilic esophagitis (EoE) is a recently recognized inflammatory disorder that needs a potential therapeutic strategy. We earlier showed that iNKT cell-deficient mice are protected from allergen-induced EoE. Therefore, we now tested the hypothesis that iNKT cells are induced in the human EoE and is a novel possible target for the treatment of human EoE. Accordingly, we examine number of iNKT cells and eosinophils and expression of iNKT-associated cell surface receptors and chemokines by performing immunofluorescence, qPCR and ELISA in the esophageal biopsies and blood samples of normal subjects (comparison control) and EoE patients. Herein, we show that iNKT cell number, their receptor subcomponents Vα24 and Vβ11 expression, and associated chemokine CXCL16 levels (or expression) are induced significantly in EoE patients compared with normal individuals. In addition, we show that CXCL16 levels (or expression) correlate with the mRNA levels of Vα24 receptor but not well with esophageal eosinophilia in human EoE. Of note, we show that *in vivo* activation of iNKT cells is sufficient to induce EoE in mice. Furthermore, we show that anti-mCD1d- and anti-hVα24Jα18-neutralizing antibody treatment protects allergen-induced experimental EoE. Taken together, we have shown first time that iNKT cells have a critical pathogenic role in human and experimental EoE. iNKT cell neutralization by humanized anti-CD1d and anti-Vα24Jα18 antibodies might be a novel and potential therapy for human EoE.

Eosinophilic esophagitis (EoE) is a chronic inflammatory disease associated with marked eosinophil infiltration in the esophageal epithelium.^1, 2, 3^ EoE is a T-cell dependent, Th2 cytokine-mediated allergic disease, and it has been shown that IL-15 is critical in the initiation and progression of EoE.^4, 5, 6, 7^ IL-15 has the ability to stimulate, proliferate and differentiate T cells and is essential for the growth and survival of CD8^+^ T cells, NK cells and iNKT cells. The iNKT cells are the non-conventional population of T cells that express a canonical invariant TCR-chain (Vα14-Jα18 for mice and Vα24-Jα18 for humans) and a TCR-chain using limited V segments (Vβ8.2 and 7 for mice and Vβ11 for humans).^8, 9, 10, 11, 12, 13^ The iNKT subsets are well characterized in health and disease^14^ and have been shown to ameliorate airway inflammation and cardiac remodeling.^15, 16^ It has been shown that an iNKT cell system recognizes the glycolipid antigen and bridges the innate and acquired immune system and used as a therapeutic target for a number of diseases including cancer.^12^ In addition, the iNKT cell activation releases a number of inflammatory Th1, Th2 and Th17 cytokines including interferon (IFN)-γ, IL-4,^17, 18, 19, 20^ IL-5 and IL-13 that are crucial regulators of inflammation.^10, 18, 21, 22^ Previously, iNKT cells were implicated in the induction of number of allergic diseases including asthma.^23, 24, 25, 26^ Recently, we reported that IL-15Rα and CD1d-gene-deficient mice are protected from the induction of experimental EoE.^27, 28^ Earlier, we and other investigators have made significant progress in understanding EoE pathogenesis; however, novel therapy for EoE is still lacking. A diet consisting exclusively of an elemental (amino acid-based) formula frequently improves symptoms and normalizes esophageal pathology;^29, 30^ however, the approach is often not tolerated and is expensive for patients. Alternatively, systemic steroids are used for acute exacerbations, whereas topical glucocorticoids are used to provide long-term control of EoE.^30, 31^ Glucocorticoid treatment shows a significant reduction in esophageal eosinophilia; however, the molecular mechanisms involved in the remission have still not been established. We earlier identified a key role for IL-5 and IL-13 in eliciting esophageal eosinophilia^6, 32^ and tissue remodeling,^33^ which provided the impetus for the current ongoing clinical trials of humanized anti-IL-5 antibody treatment for EoE.^34^ However, the long-term therapy is not promising as it was initially thought. This may be because IL-5 is only a survival factor for eosinophils and not the initiator of human EoE. Similarly, we earlier reported that *eotaxin-3* is highly induced gene in EoE patients;^35^ however, eotaxin-3 is absent in mouse genome, and we are unable to test its critical role in an EoE experimental model. Moreover, even if we try to block eotaxin-3 using neutralization anti-eotaxin-3 antibody, the possibility is that eotaxin-1 and eotaxin-2 may compensate the activity of eotaxin-3. Furthermore, the anti-IL-13 antibody therapy may not also work well in human, as we recently found that IL-13 is not critical for allergen-induced EoE.^36^ These concerns highlight the importance to uncover the other target molecules that may have a potential use in EoE therapy. We recently showed that iNKT cell-deficient mice are protected from peanut allergen-induced EoE; therefore, we focused our present investigation to examine the role of iNKT cells in human EoE. Herein, we first demonstrate that iNKT cells and their associated chemokine CXCL16 are induced in human EoE, and iNKT cell activation *in vivo* is sufficient to promote EoE in mice.

Importantly, we show that blocking iNKT cells by neutralizing anti-mCD1d or anti-hVα14Jα18 antibodies protects allergen-induced EoE in an experimental model of EoE.

Taken together, we have shown first time an upstream contribution of iNKT cells in the induction of EoE pathogenesis. iNKT cell neutralization protects both peanut and Aspergillus-induced EoE in an experimental model. In conclusion, present findings indicate that neutralizing humanized anti-CD1d and anti-Vα24Jα18 antibodies may be novel and possible potential therapeutic agents for the treatment of human EoE.

## Results

### iNKT cells are accumulated in the epithelial mucosa of EoE patients

**Figure 1:**
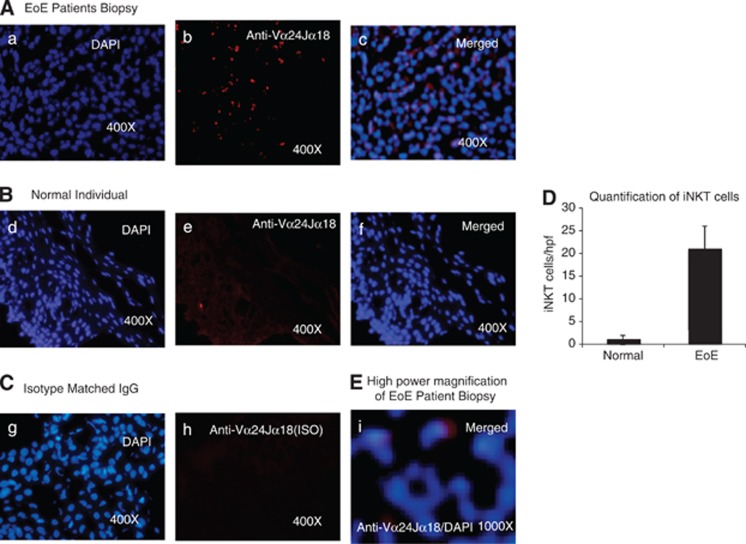
Detection of iNKT cells in the esophageal biopsies of normal individuals and EoE patients. Immunofluorescence staining of esophageal biopsies was performed by using an iNKT cell-specific receptor anti-hVα24Jα18 antibody (eBioscience) and DAPI mounting material of normal (comparison control) individuals and EoE patients. A representative photomicrograph of an EoE patient biopsy detected a number of anti-hVα24Jα18-positive cells in the esophageal biopsies of EoE patients ((**A**), original magnification × 400 photomicrograph (**b**)). The DAPI-merged photomicrograph clearly shows that the Vα24Jα18 receptors are expressed on a number of cells in the epithelial mucosa ((**A**), original magnification × 400 photomicrograph (**c**)). Esophageal biopsies of normal (comparison control) subjects had non-detectable anti-hVα24Jα18-positive cells as shown following merging with a DAPI-stained photomicrograph ((**B**), original magnification × 400 (**e**, **f**)). The esophageal biopsies of isotype-matched IgG (eBioscience) antibody did not show any positive staining in the esophageal epithelial mucosa of EoE patients ((**C**), original magnification × 400 (**h**); *n*=6 normal (comparison control) individuals and *n*=7 EoE patients). The quantitation of iNKT cells in normal (comparison control) individuals and EoE patients are shown in (**D**). The level of significance was calculated by using the Mann–Whitney test. *P*<0.0001, *n*=6–7. A high magnification photomicrograph shows cell surface staining (red) by an anti-hVα24Jα18 antibody of DAPI stained nucleus ((**E**), original magnification × 1000 (**i**)).

**Figure 2:**
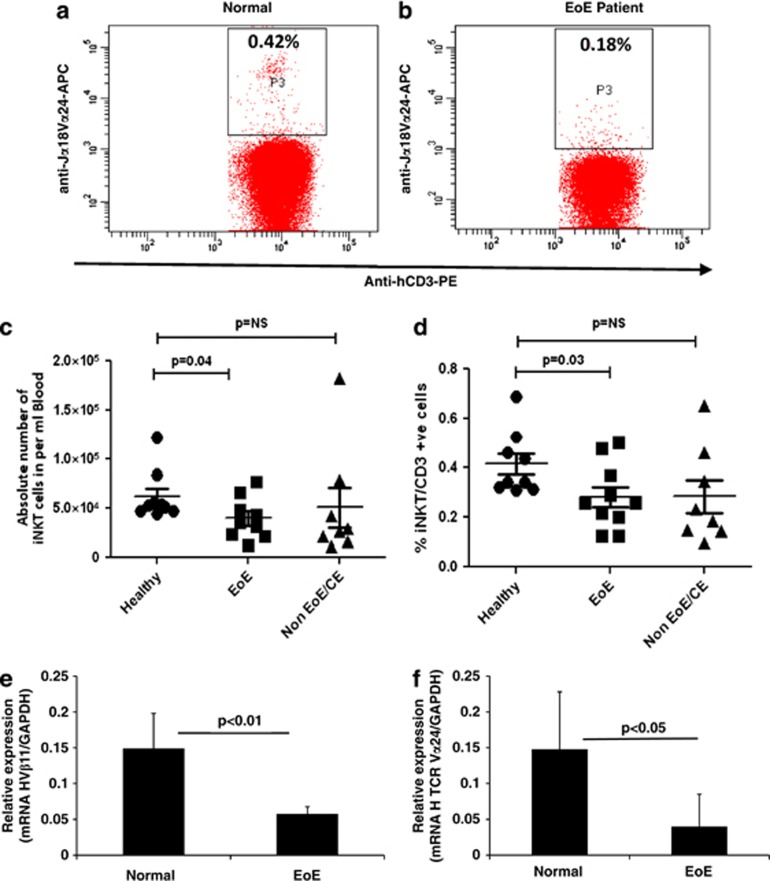
Analysis of number of iNKT cells in the blood of normal individuals and EoE patients. Flow cytometry analysis was performed to examine iNKT cells in the total blood cells using an APC-conjugated human anti-hVα24Jα18 antibody. A representative dot-plot analysis of normal (comparison control) subjects and EoE patients is shown (**a**, **b**). Total blood cells from normal subjects (**a**), or EoE patients (**b**) were analyzed for iNKT cells by gating CD3+ cells for Vα24Jα18+ utilizing isotype-matched control IgG or loaded APC-conjugated anti-hVα24Jα18 antibody. The absolute number as well as percent of iNKT cells is shown in normal (comparison control) subjects, active EoE patients and non-EoE or chronic esophagitis (CE) patients, *n*=8/subject (**c**, **d**). The levels of mRNA expression of iNKT cell-specific receptors Vβ11 and Vα24 normalized with GADPH in normal individuals and EoE patients (**e**, **f**). The level of significance was calculated by using the Mann–Whitney test.

**Figure 3:**
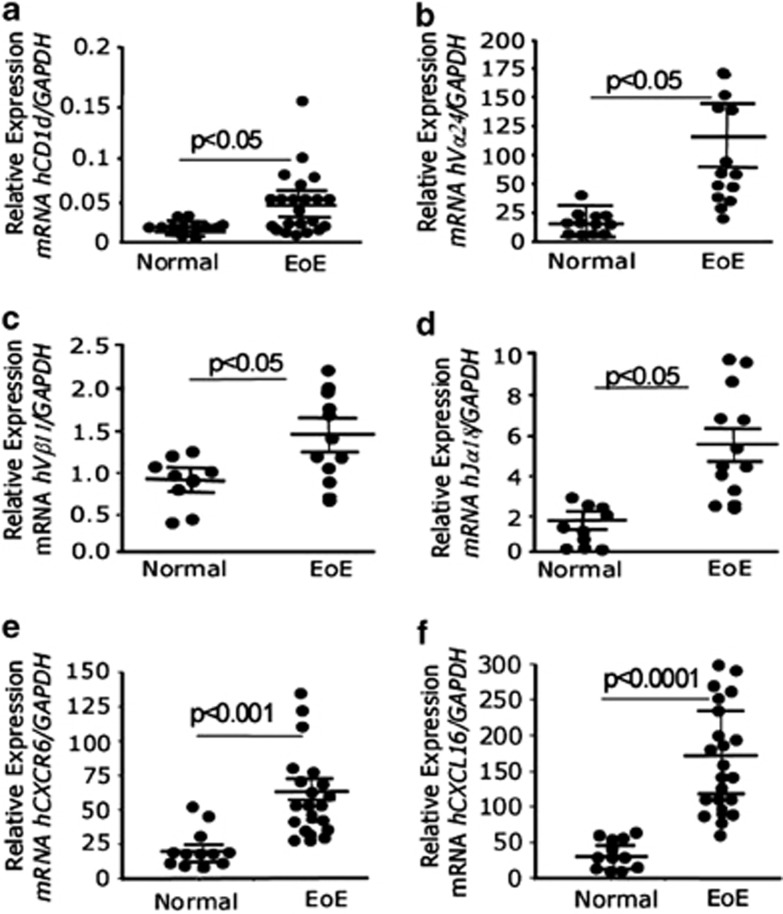
Analysis of iNKT cell-specific genes in human esophageal biopsies. The mRNA levels of iNKT cell surface molecule, TCR and T-cell components like CD1d (**a**), Vα24 (**b**), Vβ11 (**c**), Jα18, (**d**), CXCR6 (**e**) and chemokine CXCL16 (**f**) were examined by performing quantitative real-time PCR analysis. Each data point represents an individual patient (*n*=12–15 normal and 24–28 in EoE). The mRNA expression was normalized to GAPDH and expressed as relative expression. Statistical significance was calculated using the Mann–Whitney test. *P*-values for each experiment are provided in the figure (**a**–**f**).

IL-15 is known as a growth and survival factor for iNKT cells,^37, 38, 39^ and we recently reported that IL-15 mRNA and protein levels are increased and correlate with esophageal eosinophilia in patients with EoE.^27^ In order to further explore the relationship of IL-15 and iNKT cells in the pathogenesis of human EoE, we performed immunofluorescence staining on frozen proximal and distal esophageal biopsy sections of normal subjects and EoE patients, using an iNKT cell-specific receptor anti-hVα24Jα18 antibody (eBioscience, San Diego, CA, USA). Our analysis indicated that induced numbers of iNKT cell receptor, anti-hVα24Jα18, positive cells are accumulated in the esophageal mucosa of human EoE (Figure 1A). In contrast, none to few anti-hVα24Jα18-iNKT-positive cells were detected in esophageal biopsies of normal subjects (Figure 1B). The specificity of the staining was demonstrated by the lack of positive staining of isotype-matched control antibodies on the EoE patient biopsy sections (Figure 1C). Further, anti-hVα24Jα18-positive iNKT cells were quantified in the esophageal biopsies of normal subjects and EoE patients. The analysis indicated that ∼20±5 iNKT cells (Vα24Jα18+) accumulated per high power field (hpf) in the esophageal biopsies of EoE patients compared with average <1 iNKT cells/hpf (Figure 1D). In addition, we examined the number of circulating iNKT cells in normal subjects (comparison control individual), EoE and non-EoE/CE patients by staining total blood cells with anti-CD3 and anti-hVα24Jα18 antibodies followed by flow cytometry analysis, and gated populations are shown in Figures 2a–d. The absolute number as well as percent of iNKT cells was decreased in the blood of EoE patients compared with normal subjects or non-EoE/CE patients (Figures 2e and f). The decrease of iNKT cells was confirmed by examining the mRNA level of iNKT cell specific T-cell components in the blood of normal and EoE patients. The levels of Vβ11 and Vα24 were found significantly reduced in EoE patients compared with normal (comparison control) subjects (Figures 2g and h). All EoE patients used in the analyses have active EoE (>15 eosinophil/hpf) without any steroids or other treatments. The decreased number of iNKT cells in the blood of human EoE patients may be due to the induced chemotaxis of iNKT cells in the esophagus. Notably, our flow cytometric analysis of comparison control normal subjects exhibit a large inter-donor variation of iNKT cell percent as well as in absolute cell number counts that suggests iNKT cell response varies from individual to individual even at healthy state. However, in the earlier studies investigators indicated that iNKT cells are rarely present in normal individuals.^14^ To further confirm the anatomical relationship of iNKT cells with esophageal epithelial cells, we measured mRNA levels of iNKT cell-specific genes in the esophageal biopsies from normal subjects and EoE patients. The quantitative real-time PCR analysis indicated that mRNA expression for several iNKT cell-related genes such as cell surface molecule, TCR and their subcomponents, CD1d, Vα24, Vβ11, CXCR6, and chemokine CXCL16 were significantly induced in the esophageal biopsies of EoE patients compared with comparison control normal subjects (Figures 3a–f). The details of primers used to analyze the mRNA levels of iNKT cell-associated genes are provided in Supplementary Table 1.

### Esophageal epithelial cells are the source of iNKT cell-associated chemokine CXCL16

**Figure 4:**
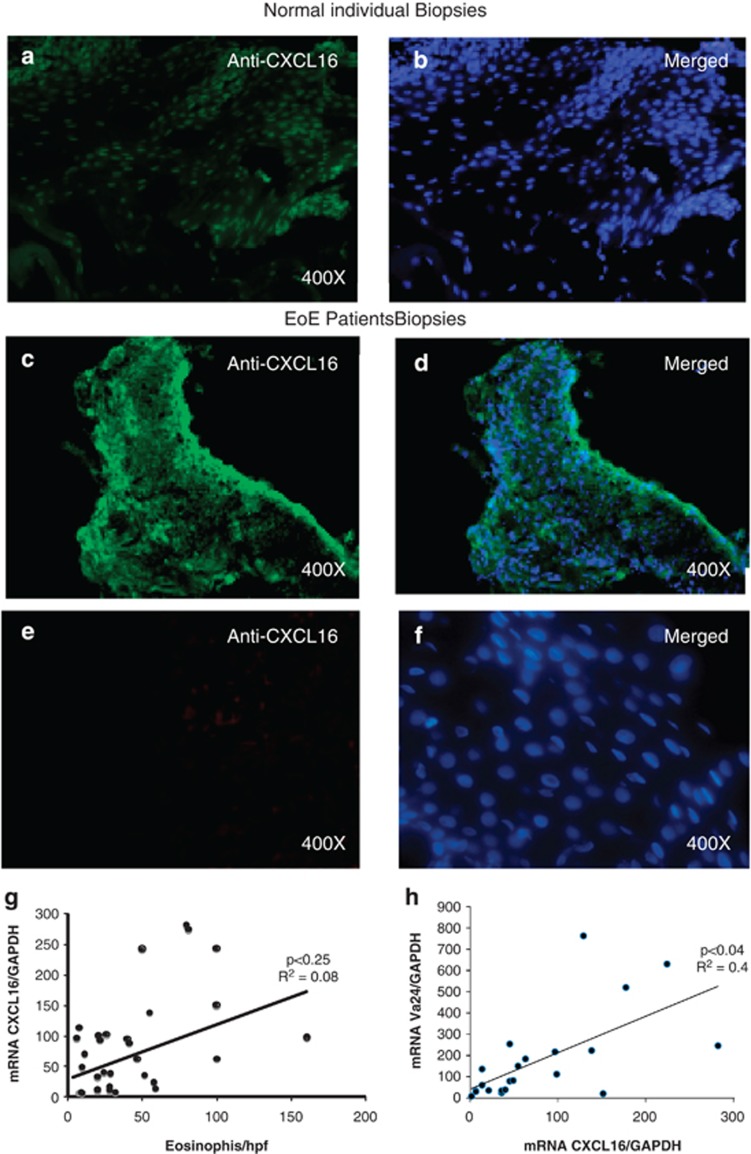
Analysis of CXCL16 expression in human esophageal biopsies. A representative photomicrograph of baseline esophageal CXCL16 expression in normal (comparison control) subjects (**a**, **b**) and EoE patients (**c**, **d**) stained with anti-CXCL16 antibody (**a**–**d**) or isotype-matched IgG (**e**, **f**) and overlapped with a DAPI-stained mounted reagent. Correlations between the peak eosinophil number/hpf vs CXCL16 mRNA expression and Vα24 vs CXCL16 mRNA expression normalized with GADPH in human EoE (**g**, **h**). The *r-*value was calculated using the Spearman correlation test (*n*=24–28). Statistical significance was calculated using both the Mann–Whitney and Kruskal–Wallis test.

Murine and human iNKT cells home in the tissue via interaction of receptor CXCR6 and ligand CXCL16;^40, 41^ therefore, we next examined the source of CXCL16 in the esophageal mucosa by performing rabbit anti-CXCL16 immunofluorescence staining of the esophageal biopsies of comparison control normal subjects and EoE patients. The esophageal mucosa of normal subjects had minimal or undetectable CXCL16 protein expression (Figures 4a and b) compared with esophageal epithelial cells of EoE patient biopsies that had increased expression of CXCL16 protein (Figures 4c and d). The DAPI mounting material stain the cell nucleus and the merged photomicrograph clearly indicated that anti-CXCL16 antibody-stained cells are present in the epithelial mucosa of the biopsies. Esophageal biopsies with isotype-matched IgG (primary anti-CXCL16 antibody is replaced by rabbit IgG isotype matched), the isotype-matched IgG antibody did not show any positive staining in the mucosa of normal or EoE patients (Figures 4e and f). Furthermore, a weak positive correlation was observed with CXCL16 mRNA expression with the mRNA levels of iNKT cell-associated receptor Vα24 and intraepithelial eosinophil counts in the esophageal mucosa of EoE patients (Figures 4g and h). However, the correlation of both the genes was not found statistically significant.

### iNKT cells are increased in a mouse model of EoE, and iNKT cell-deficient (*CD1d*-null) mice are protected from the induction of experimental EoE

Next, we examined iNKT cell numbers by flow cytometry in total isolated esophageal cells from mice following allergen-induced experimental EoE or saline control treatment. Our analysis indicated that NK (CD3^−^DX5^+^) cells and CD1d-αGalCer-tetramer^+^ iNKT cells are increased in the esophagus. The strategy for gating NK or iNKT cells from isolated esophageal cells by flow cytometeric analysis is shown (Supplementary Figures 1A–F). The anti-CD3/anti-DX5 antibody will identify NK cells and CD1d-αGalCer tetramer will identify iNKT cells. The CD1d-αGalCer tetramer staining shown here is performed after collagenase digestion of esophageal tissue (Supplementary Figures 1D–F).^42^ Total cells isolated after collagenase digestion from the esophagus provided some acceptable iNKT cell analysis by CD1d-tetramer staining. Although, there is a possibility that enzymatic tissue digestion might alter the CD1d tetramer binding epitope of iNKT cells and limits the detection of iNKT cells by flow cytometry but we detect iNKT cells as a scattered populations by flow cytometry. Notably, scattered CD1d-αGalCer+ iNKT cell population was not detected in normal subjects, confirms that these are iNKT cells. The CD1d gene-deficient mice challenged with *Aspergillus* extract were used as a control for iNKT cell analysis. Notably, our analysis couldn't detect any iNKT cells in the esophagus of these mice (data not shown). The mean NK cell (CD3−/DX5+) numbers increased from 3.9±1.4 × 10^3^/esophagus to 8.3±1.1 × 10^3^/esophagus (mean±s.d., *n*=3 experiments) (Supplementary Figure 2A). The mean esophageal CD3+/CD1d-αGalCer-tetramer+ cell levels were 1.8±0.9 × 10^3^/esophagus in saline-challenged and 4.1±0.7 × 10^3^/esophagus in allergen-challenged mice (mean±s.d., *n*=3 experiments) (Supplementary Figure 2B). We also examined the iNKT cell-associated chemokine CXCL16 by quantitative real-time PCR analysis in saline- and allergen-challenged mice and found that CXCL16 expression is induced in the esophagus of mice following the induction of experimental EoE (Supplementary Figure 2C). iNKT cells express CXCR6; therefore, induced CXCR6 expression in experimental EoE is also observed (Supplementary Figure 2D). Furthermore, in order to define the role of iNKT cells in Aspergillus-induced EoE, we induced experimental EoE in iNKT cell-deficient (CD1d-null) mice following the previously described Aspergillus-challenged protocol.^7^ This mouse model of allergic EoE have features of most of the characteristics of human EoE, such as intraepithelial eosinophils, eosinophil microabsesses, epithelial cell hyperplasia and extracellular eosinophilic granules.^7^ The CD1d-null mice were protected from eosinophil accumulation in the esophagus compared with wild-type mice following the induction of allergen-induced experimental EoE (Supplementary Figure 2E). The number of eosinophils in the esophagus of allergen-treated wild-type mice was 58.1 × 15.6mm^−2^ compared with 5.1 × 0.7mm^−2^ (mean s.d., *n*=12, *P*<0.001) in saline-treated mice. However, the eosinophils in the esophagus of allergen-treated CD1d-null mice were 10.2 × 5.3mm^−2^ compared with 3.8 × 0.5mm^−2^ (mean s.d., *n*=12) in saline-treated mice. While the number of eosinophils in the lungs of allergen-challenged CD1d-null mice was only modestly reduced compared with the allergen-challenged wild-type and saline-challenged mice, the difference was statistically significant (Supplementary Figure 2F).

### iNKT cell activation is sufficient to induce EoE in mice

**Figure 5:**
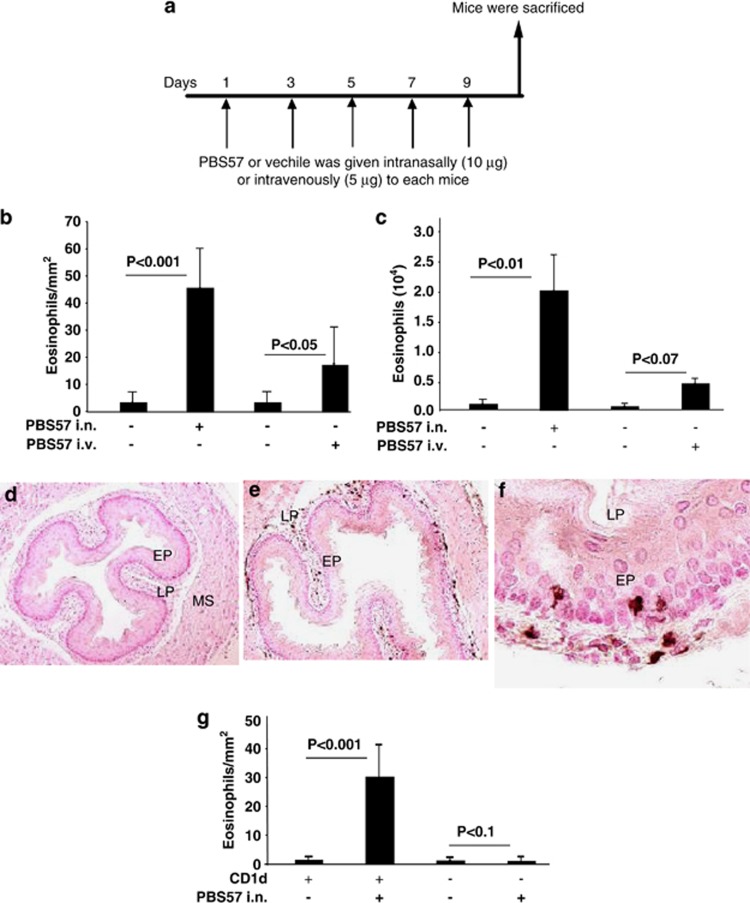
*In vivo* iNKT cell activation by PBS57 and esophageal eosinophil analysis. Mice (BALB/c) were exposed to repeated exposure of intravenous (i.v.) or intranasal (i.n.) PBS57  as per the protocol shown in (**a**). After 20–24 h of the last PBS57 or vehicle challenge, mice were killed, and eosinophil levels were evaluated in the esophagus (**b**) and BALF (**c**). Representative anti-MBP antibody-immunostained high-resolution photomicrographs of intraepithelial eosinophils in the esophagus after vehicle (**d**, original magnification × 100) or intranasal PBS57 challenge (**e**, original magnification × 100 and **f**, original magnification × 400). The absolute numbers of eosinophils in the esophagus of CD1d-null mice and wild-type mice after intranasal PBS57 challenge of mice (**g**). Data are expressed as mean±s.d., *n*=12 mice/group. EP, epithelium; LP, lamina propria; MS, muscularis mucosa.

We next tested whether iNKT cell activation is sufficient to induce EE in mice. Accordingly, we delivered iNKT cell-specific agonist αGalCer analog PBS57^43^ to naive mice as per the protocol shown (Figure 5a). An intense eosinophilia was detected in the esophagus along with moderate eosinophilia in the lung of mice given intranasal and intravenous PBS57 (Figures 5b and c). The intranasal PBS 57-challenged mice accumulated intraepithelial eosinophils in the esophageal epithelium (Figures 5d and e). The mice given intranasal or intravenous vehicle control did not have detectable levels of eosinophils in the esophageal mucosa (Figure 5f). To validate that *in vivo* PBS57 treatment is specific to iNKT cells, we treated CD1d-null mice with intranasal PBS57. The PBS57-treated CD1d-null mice did not induce EoE (Figure 5g).

### Human iNKT cell activation *in vitro* by PBS57 induces STAT5-associated Th2 cytokine

PBS57 is a known stimulator of iNKT cells,^43^ and we have shown that intranasal delivery of PBS57 alone promotes EoE in mice (Figures 5d and e). Therefore, we tested the hypothesis that human iNKT cells^44^ activated by PBS57 would induce eosinophil active Th2 cytokines. Accordingly, we treated human iNKT cells with different doses of PBS57. A dose-dependent increase in the mRNA and protein levels of the Th2 cytokines IL-4 (Supplementary Figure 3A, B), IL-5 (Supplementary figure 3C, D) and IL-13 (Supplementary Figure 3E, F) was detected in iNKT cells and their culture supernatant, respectively, following 24 h of PBS57 treatment. In addition, flow cytometric analysis was performed to determine whether Th2 cytokine induction by PBS57-treated iNKT cells is associated with the activation of the STAT family of molecules. The analysis revealed that STAT5 was phosphorylated in PBS57-treated iNKT cells within 2–8 min, whereas no STAT6 phosphorylation was observed at any time point following PBS57 treatment (Supplementary Figure 3G).

### Pharmacologically delivered neutralizing anti-CD1d and anti-human Vα24Jα18 antibodies protects both peanut and Aspergillus-induced EoE in an experimental model of EoE

**Figure 6:**
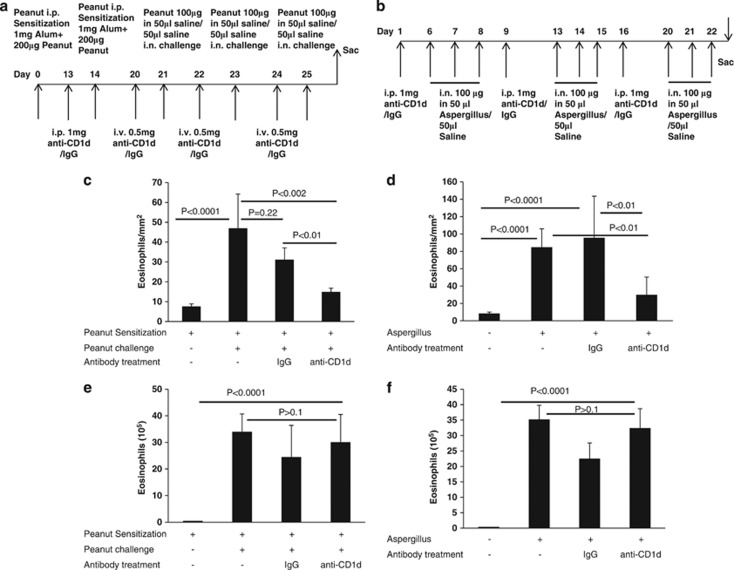
iNKT neutralization protects mice from food- and aeroallergen-induced experimental EoE. The Balb/c mice were subjected to iNKT cell neutralization by injecting the anti-CD1d monoclonal antibody or isotype-matched IgG in an allergen-induced mouse model of EoE as per the protocol (**a**, **b**). The number of eosinophils in the esophagus was analyzed in the food allergen-sensitized mice treated with anti-CD1d or IgG and challenged with saline (−) or peanut (+) is shown following 24 h after last saline or peanut challenge (**c**). Data are expressed as mean±s.d., *n*=9–12 mice/group. The anti-CD1d or IgG treated and intranasally challenged with saline (−) or aeroallergen (*Aspergillus fumigatus; +*) were also examined for esophageal eosinophilia and are shown as eosinophils/mm^2^ following 20 h after last Aspergillus or saline challenge (**d**). Data are expressed as mean+s.d., *n*=12 mice/group. The eosinophil numbers in bronchoalveolar lavage fluid (BALF) were counted in food allergen-sensitized mice treated with anti-CD1d or IgG and challenged with saline (−) or peanut (+) and CD1d-treated and challenged with saline or Aspergillus (**e** and **f**). Statistical significance was calculated using both the Mann–Whitney and Kruskal–Wallis test, and the significance levels are shown in respective figures.

Our investigation showed that iNKT cell numbers are elevated in the esophageal biopsies of EoE patients. Therefore, we tested the hypothesis whether iNKT cell neutralization protects allergen-induced EoE. Accordingly, mice challenged with saline, peanut or Aspergillus were treated with an anti-CD1d antibody or isotype-matched IgG mice as per the protocol (Figures 6a and b). There was significant number of eosinophils compared with saline-challenged mice. However, anti-CD1d antibody treatment significantly reduced the esophageal eosinophila in allergen (both peanut and Aspergillus)-challenged but not in saline- or IgG-challenged mice (Figures 6c, and d). In contrast, anti-CD1d treatment has no effect on airway eosinophilia in allergen (both peanut and Aspergillus)-challenged mice compared with saline or IgG treated mice (Figures 6e and f).

Further, we also tested an anti-hV24J18 neutralizing antibody in our allergen (both peanut and Aspergillus)-induced experimental EoE model. We used anti-hV24J18 because this antibody has subcomponents of the mouse iNKT cell receptor Jα18 and may recognize and bind with mouse iNKT cells. If it works then we can propose this antibody for human clinical trial for EoE therapy in patients. As expected the anti-hV24J18 neutralizing antibody also significantly protects Aspergillus-induced EoE and show reduced esophageal eosinophilia in peanut-induced EoE (Supplementary Figures 4A–D).

Furthermore, we also tested an iNKT cell-associated chemokine anti-CXCL16 neutralizing antibody to test whether blocking of CXCL16 chemokine also protect allergen-induced EoE in mice. Surprisingly, neutralizing antibody of iNKT-associated chemokine CXCL16 treatment fail to protect allergen-induced experimental EoE in mice (data not shown). Taken together, our data indicate that iNKT neutralization either by anti-CD1d or by anti human-V24J18 treatment protect allergen-induced EoE in mice.

## Discussion

EoE is a recently recognized disease by the medical community involving mucosal eosinophilia, which is differentiated from gastroesophageal reflux by the lack of response to acid suppression.^45, 46^ The identification and prevalence of EoE is rising throughout the world.^47^ While consensus guidelines for diagnosis were recently recommended,^48^ a novel effective therapy for EoE has yet to be established. The present study demonstrated a novel role of iNKT cells in the pathogenesis of EoE. Here, we provide evidence that iNKT cells are induced in the esophageal biopsies of EoE patients compared with normal subjects assessed by immunostaining with a series of the iNKT cell-specific cell surface markers/receptors CD1d, Vβ11, Vα24 and Jα18. These findings and our earlier report^28^ indicate that iNKT cells may be involved in EoE pathogenesis and are critical in the induction of peanut-induced experimental EoE. Furthermore, a low level of mRNA and protein expression of the receptors Vα24 and Jα18 in the esophagi of normal individuals indicates that very few iNKT cells reside in the esophagus at baseline in healthy conditions. Notably, we found that iNKT cells and its associated receptor V11 and V24 mRNA levels in the blood are decreased (*P*<0.01 or *P*<0.05). The decrease in blood iNKT cells may be due to the induced chemotaxis of iNKT cells in the esophageal mucosa, as iNKT cell-associated chemokines are increased in the esophageal mucosa of EoE patients. This possibility is supported by the data that iNKT cell-expressed CXCR6 specifically binds to the chemokine CXCL16 and attracts iNKT cells to the tissue.^40, 49, 50^ Here, the data demonstrate that the mRNA and protein expression of CXCR6 and its ligand CXCL16 are increased in human EoE as compared with the low baseline expression in normal individuals. Therefore, it is possible that CXCL16 and CXCR6 association is important in the recruitment of iNKT cells into the esophageal mucosa. Previously, CXCL16 was shown as a unique marker for Crohn's disease,^51, 52^ and our data suggest that CXCL16 may also be important in the pathogenesis of EoE. We show that esophageal epithelial cells are the source of CXCL16 in EoE patients. Previously, it has been shown that blocking of CXCL16 restricts chemotaxis of iNKT cell homing into the tissue;^49^ and most recently it has also been shown that CXCL16 has a critical role in iNKT cells accumulation in intestine.^53, 54^

In addition, we have demonstrated previously iNKT cell-deficient CD1d-null mice were protected from food allergen-induced EoE, hence, providing evidence of the role of iNKT cells in EoE pathogenesis.^28^ In the present study, we have confirmed that a similar mechanism is operational also in aeroallergen-induced EoE. In addition, we also show that intranasal and intravenous delivery of iNKT cell-specific agonist, αGalCer analog PBS57, to naive mice promotes EoE. This study further supports our hypothesis that iNKT cells may be the major contributor in the pathogenesis of EoE. The induction of EoE by PBS57 exposure of mice establishes that EoE pathogenesis may be a CD1d-restricted iNKT-cell responses. This notion was confirmed by observation that CD1d-null mice were protected from development of experimental EoE with PBS57, as shown in Figure 5. Of note, mechanistically we show that iNKT activation produces Th2 cytokines, which is in accordance with the earlier reports that mature iNKT cells are a prominent source of Th1 and Th2 cytokines.^17, 18^ We further report that Th2 cytokines produced by PBS57-activated iNKT cells are regulated by STAT5 and independent of STAT6 (Supplementary Figure 3G and H). Our present and previous studies now provide sufficient evidence that iNKT cells promote EoE. Our findings are in accordance with our previously reported studies where we have shown that IL-15 activates only a specific subpopulation of CD4^+^ T cells to release Th2 cytokines via the STAT5 pathway,^27^ and T-cell-deficient mice were completely protected from disease induction.^55^ We have also reported that conventional T-cell subset CD8-T-cell-deficient mice were not but CD4-T cell-deficient mice were only partially protected from disease induction. The CD4 antigen is expressed not only by class II major histocompatibility complex (MHC)-restricted CD4^+^ T cells but also in the CD1d-restricted iNKT cells, which have both CD4^+^ and CD4^−^ subsets.^56^

**Figure 7:**
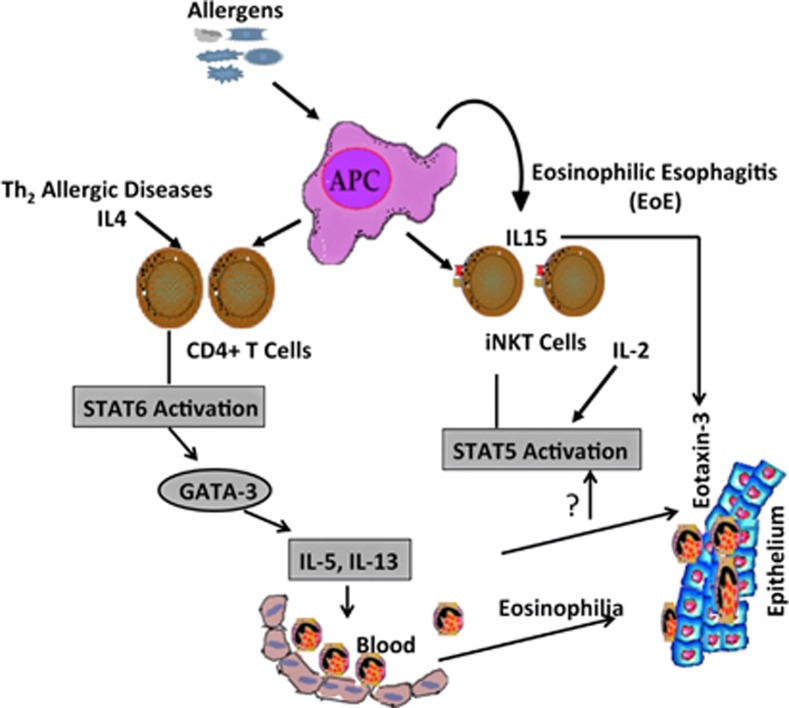
Diagrammatic representation of proposed pathway of iNKT cell-induced EoE. Allergen taken by antigen-presenting cells (APCs) is offered to the conventional (CD4+ T cells) and non-conventional T cells (iNKT cells). On the basis of our previous and current data in the manuscript, we propose that both conventional and non-conventional T cells produce eosinophil active Th2 cytokines that are regulated by signal transducer and activator of transcription (STAT) family of molecules. The induction and activation of iNKT cells along with eosinophil active cytokines, and eotaxin-3 in esophageal epithelial mucosa accumulates eosinophils into the esophagus. This cartoon model summarizes the mechanism that STAT5 regulates iNKT cell-induced eosinophilic esophageal inflammation in EoE; whereas STAT6 regulates other allergic Th2 cytokines-associated eosinophilic disorders.

Given that iNKT cell accumulation in human esophageal mucosa and their ability to secrete abundant eosinophil active cytokines (Th2 cytokines) suggest their role in the induction of Th2 cytokine-driven EoE pathogenesis.^17, 18^ Recent literature shows that milk-derived sphingomyelin and/or its related lipids potentially activate iNKT cells in children with food allergy.^57^ In addition, allergen-derived glycolipids from antigen presenting cells (APCs) are also the candidates for iNKT cell activation.^35^ Hence, these iNKT activation-mediated pathways may be involved in human EoE. Importantly, the present findings that CD1d is a critical molecule that is required for the food- and allergen-induced EoE pathogenesis, and CD1d-null mice do not induce EoE when challenged with iNKT cell ligand αGalCer analog PBS57 compared with wild-type mice support this notion. These data confirmed that induced iNKT cell may have a critical role in the pathogenesis of EoE. Our findings prompted us to hypothesize that iNKT cell neutralization may protect EoE in humans. Therefore, we conducted additional experiments to show that the iNKT cells are critical, and their neutralization by using anti-CD1d or anti-hVα24Jα18 neutralization antibodies protects food- and aeroallergen-induced experimental EoE murine models. Herein, we first time present the data that iNKT cell neutralization indeed protects peanut-and Aspergillus-induced experimental EoE. Of note, the level of allergens-induced EoE protection by an anti-hVα24Jα18 neutralization antibody is less than that by the anti-CD1d, this may be because it is an anti-human antibody and not completely blocks the mouse iNKT cell responses in experimental EoE. We also show that by simply neutralization of iNKT cell-associated chemokine CXCL16 is not sufficient to block induction of EoE. Although, CXCR6 and CXCL16 are induced in the esophageal biopsy of EoE patients and experimental EoE mouse model. This indicates that iNKT cell recruitment in the esophagus not only through CCR6 and CXCL16 interaction but some other alternate pathway is also operational. On the basis of these findings, we now finally summarized the mechanistic pathway that is operational in the pathogenesis of EoE (Figure 7).

Taken together, these studies provide evidence that CXCL16 is not critical in iNKT cell recruitment and some other chemokines and iNKT cell receptor interaction mechanism is operational in the esophagus. However, we confirm that iNKT cell neutralization protects EoE induction, at least in the experimental setup. The current EoE therapy is based on food elimination; anti-inflammatory treatments restricted to the use of glucocorticoids and humanized anti-IL-5 antibody treatment.^31, 58^ The anti-IL-5 antibody therapy is promising but their preliminary results in clinical trials are not as encouraging as hoped. This may be due to the fact that IL-5 is a surviving factor for eosinophils and not an initiator of EoE. Performing flow cytometric analysis using spleenocytes of anti-CD1d-antibody treated and non-treated allergen-challenged mice showed that iNKT cells were completely depleted following anti-CD1d antibody treatment in mice (data not shown). Our present study provides the evidence that anti-CD1d and anti-hVα24Jα18 neutralizing monoclonal antibodies may be a possible potential therapeutic target for human EoE. On the basis of these findings, we propose to conduct a double-blinded clinical trial using humanized neutralizing anti-CD1d and anti-hVα24Jα18 antibodies at multiple centers to establish a novel therapy for human EoE.

## Methods

### Patient biopsies

Formalin-fixed, paraffin-embedded biopsy samples were obtained from the esophagus of normal individuals or EoE patients as per an Institutional Review Board (IRB)-approved protocol. The comparison control normal subjects (non-EoE), chronic esophagitis (CE) and EoE patients were selected without regard to age, atopic status or gender. Diagnosis was established based on the maximum eosinophil count per high power field (hpf) ( × 400) control individuals (non-EoE or CE) was defined as having 0 esophageal eosinophils/hpf and no basal layer expansion. The normal biopsies were obtained from patients who showed symptoms typical of gastroesophageal reflux disease (GERD) and EoE but were found to have completely normal esophageal endoscopic and microscopic analyses. Typically, these patients had abdominal pain, and some had allergic diseases including asthma or rhinitis. Patients with EoE were defined by having ⩾15-esophageal eosinophils/hpf. Patients included in this study had EoE and other allergic diseases such as asthma or atopic dermatitis. All samples were used according to the patients' consent, and IRB approved protocol 2012.

### Mice

Specific pathogen-free BALB/c mice were obtained from the Jackson Laboratory (Bar Harbor, ME, USA). CD1d-null mice (original strain C129S2-Cd1^tm1Gru/^J) backcrossed to BALB/c were obtained from Dr Mattner's laboratory at Cincinnati Children's Hospital Medical Center (CCHMC).^44^ All the experiments were performed on age- and gender-matched mice 6–8 weeks of age. The mice were maintained in a pathogen-free barrier facility, and animals were handled according to institutional review board approved guidelines.

### Experimental EoE

A mouse model of allergic EoE was established using methods described previously.^7, 59^ In brief, mice were lightly anesthetized with isoflurane (Iso-Flo; Abbott Laboratories, North Chicago, IL, USA), and 100 μg (50 μl normal saline) of *Aspergillus fumigatus* (Greer Laboratories, Lenoir, NC) or 50 μl of normal saline alone was given intranasal using a micropipette with the mouse held in the supine position. In addition, we also induced experimental EoE by sensitizing the mice at 0 and 14 days with peanut 200 μg and 1 mg Alum and then challenged with 100 μg peanut on days 21, 23 and 25. After three treatments per week for 3 weeks, mice were killed between 20 h and 24 h after the last intranasal allergen or saline challenge.

### Bronchoalveolar lavage fluid

The mice were killed by CO_2_ inhalation. Immediately thereafter, a midline neck incision was made, and the trachea was cannulated. The lungs were lavaged two times with 1.0 ml PBS containing 1% FCS and 0.5 mM EDTA. The recovered BALF was centrifuged at 400 × *g* for 5 min at 4 °C and resuspended in PBS containing 1% FCS and 0.5 mM EDTA. Total cell numbers were counted with a hemocytometer. Cytospin preparations of 5 × 10^4^ cells were stained with Giemsa-Diff-Quick (Dade Diagnostics, Aguada, PR, USA), and differential cell counts were determined. The BALF eosinophil counts were expressed as an indication of lung eosinophilia.

### Immunohistochemical analysis

5 μm esophageal paraffin tissue sections of mouse esophagus were immunostained with antiserum against mouse eosinophil major basic protein (anti-MBP antibody, bought from Mayo Clinic, Scottsdale, AZ, USA) as per the method described previously.^7, 55, 59^ Eosinophils were quantified by counting the MBP positive-stained cells in each tissue section with the assistance of digital morphometry using the Metamorph Imaging System (Universal Imaging Corp, West Chester, PA, USA) and expressed as eosinophilsmm^−2^ tissue area as described earlier.^7, 27, 59^ The eosinophils in non-EoE and EoE patient esophageal biopsies were identified in eosin- and hematoxylin-stained tissue sections and quantified as eosinophils/hpf ( × 400).

### Immunofluorescence staining

Cryostat sections from frozen esophageal biopsies of normal subjects (non-EoE) and EoE patients were fixed, blocked with normal goat serum to reduce non-specific binding, and incubated with an anti-hVα24Jα18 antibody (eBioscience) to stain iNKT cells and anti-CXCL16 antibody (kind gift from Koji Sayama, Ehime University School of Medicine, Shitsukawa, Toon-city, Ehime, Japan). The iNKT and CXCL16 immunostaining was performed as per the protocol reported earlier^60^ Images were captured using an Olympus BX51 microscope with appropriate filters. Anti-hVα24Jα18-positive cells were counted on each stained tissue sections per high power field ( × 400) and expressed as ‘number of cells/hpf.' Total of 4–5 high power fields in each esophageal sections was evaluated in EoE or normal control individual biopsies sections.

### Human iNKT cell line

The non-immortalized human iNKT cell line^61^ was provided by Dr Mattner (CCHMC, Cincinnati, OH) and was grown in RPMI-1640 (HyClone Laboratories, Logan, UT, USA) and Click's Medium (Sigma Aldrich, St Louis, MO, USA) containing 10% fetal calf serum (FCS, Gibco BRL), 1% 200 mM L-Glutamine (Invitrogen, Grand Island, NY, USA) and penicillin-streptomycin (Gibco BRL). The cells were grown and split into six-well plates starting with a concentration of 1 × 10^6^ cells in 2 ml media per well. Subsequently, different concentrations of the iNKT cell-specific agonist alpha-GalCer analog PBS57 was added (0, 100, 500, 100 ng ml^−1^) to the culture medium and collected following 24 h for the mRNA and protein levels of eosinophil active Th2 cyokines. iNKT cell-induced activation of pSTAT5 and pSTAT6 was also determined by exposing iNKT cells to different concentrations of PBS57.

### Flow cytometry and antibodies

The total blood or isolated esophageal cells was stained with cell surface molecule-specific antibodies for flow cytometer analysis. Total esophageal and blood cells were isolated as per the protocol described earlier.^42^ The following reagents were used for specific antigen analysis: anti-CD3, anti-CD4, anti-CD45, anti-DX5, anti-Vα24Jα18 and their respective isotype controls obtained from eBiosciences. Human and mouse CD1d-tetramer was obtained from the tetramer core facility of the National Institute of Allergy and Infectious Diseases. FcR block (anti-CD16/CD32 Ab) was added to all surface staining mixtures. 7AAD was used to exclude dead cells. Anti-pSTAT5, and pSTAT6 antibodies were also used to examine specific STAT phosphorylation with respective isotype antibodies controls. The cells were incubated for specific antigens with the required combination of antibodies at 4 °C for 45 min followed by two washes. FACS analysis was performed using a FACSCalibur (BD Biosciences, San Jose, CA, USA) and analyzed using CellQuest software (BD Biosciences).

### PBS57-induced experimental EoE in mice

Five doses of 10 μg of PBS57 or vehicle were given intratarcheally or 5 μg PBS57 intravenously to each mouse on alternate days. After five treatments, mice were killed between 22 h and 24 h after the last intratracheal or intravenous challenge and analyzed for esophageal and lung eosinophilia following anti-MBP antibody staining or BALF analysis respectively.

### iNKT neutralization using anti-CD1d and anti-hVα24Jα18 antibodies in experimental EoE

The experimental EoE is developed by challenging the mice with Aspergillus as described above and anti-CD1d (eBiosciences) or anti-hVα24Jα18 antibodies (eBiosciences) treatment was done each week once for 3 weeks as shown in the protocol figures. Similarly, peanut allergen-induced EoE was developed in mice as described above, and anti-CD1d or humanized anti-hVα24Jα18 antibodies treatment was done in peanut sensitized and challenged mice as shown in the protocol of their respective figures.

### Statistical analysis

For all cell counts, stained slides were analyzed randomly and in a blinded manner. For non-parametric data, the Mann–Whitney U-test was employed for comparison between two groups, and the Kruskal–Wallis test was used for comparisons among three or more groups. Parametric data were compared using *t*-tests or analysis of variance. Values are reported as mean±s.d. *P-*values <0.05 were considered statistically significant.
