# Supplementary material for: Invariant natural killer T-cell neutralization is a possible novel therapy for human eosinophilic esophagitis
Source: Clin Transl Immunology. 2014 Jan 10;3(1):e9–. doi: 10.1038/cti.2013.13 (PMC4232063; doi:10.1038/cti.2013.13)
Supplement: Supplementary Information [file cti201313x1.pdf]

Supplementary Table 1. List of primer sequences used for qPCR

Genes	Sense and anti-sense primer sequence
hIL-4	5’-AACAGCCTGACAGAGCAGAAGA<br>5’-GCCCTCCAGAAGGTTTCCTT
hIL-5	5’-GCTTTGCATTTGAGTTTGCTAGCT<br>5’-TGGCCGTCAATGTATTTCTTTATTAAG
hL-13	5’-ACAGCCCTCAGGGAGCTCAT<br>5’-TCAGGTTGATGCTCCATACCAT
hCXCL16	5□-GGCCCACCAGAAGCATTTAC<br>5□-CTGAAGATGCCCCCTCTGAG
hCXCR6	5′-ATGCCATGACCAGCTTTCACT<br>5′-TTAAGGCAGGCCCTCAGGTA
mCXCL16	5’-TGCCAGGCGATGGCAACCAG<br>5’-GTCCAGGGGGTGCTCGTGTCC
hMICB	5’-TCTGCTATGCCATGTTTTGT<br>5’-GCCCTCAGTGGAACCAGTGGA
hCD1d	5'- GCCTGTCCTGTCGGGTGAAGC<br>5'- GCTGTTCCTCCTCATTGTGGGCT
hTCRVα24	5’-GAAAGTTTAGGTTCGTATCTGTTTCA<br>5’-GATATACAGCAACTCTGGATGCA
hTCRVβ11	5’-TCAACAGTCTCCAGAATAAGGACG-3’<br>5’-GTGGGAGATCTCTGCTTCTG-3’
hTCRJα18	5’-GTGGGAGATCTCTGCTTCTG-3’<br>5’-GGATATCAGGCCAGACAGTC-3’
hGADPH	5’TGGAAATCCCATCACCATCT3’<br>5’GTCTTCTGGGTGGCAGTGA T3’

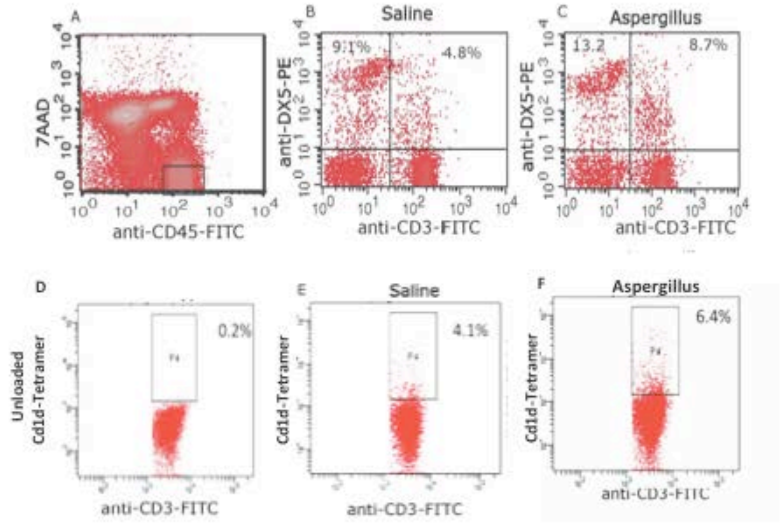

Supplementary figure 1. Gating Strategy and analysis to examine NK anf iNKT cells. Fluorescent-activated cell sorter (FACS) analysis was performed to examine activated NK and iNKT cells in the total esophageal cells isolated from enzymatic digestion of the esophagus. The total esophageal cells from saline- and allergen (*Aspergillus*)-challenged mice were stained with anti-CD45 (panmarker), anti-CD3ε (total T cells) and anti-Vα24Jα18 (iNKT cells), anti-DX5 (NK cells) and 7AAD (live cells). Total live leukocytes (CD45+/7AAD-) were gated and analyzed for NK cells (A). A representative dot-plot analysis of saline- and allergen-challenged esophageal cells is shown for induced NK cells by anti-CD3ε/-DX5+ and anti-CD3ε/ anti-DX5- gating (B, C). Furthermore, A representative dot plot analysis for iNKT cells are also shown by using anti-CD3ε/CD1d-tetramer (unloaded) (D), saline- and allergen challenged mice loaded with antiCD3ε/CD1d-aGalCer-tetramer (E, F).

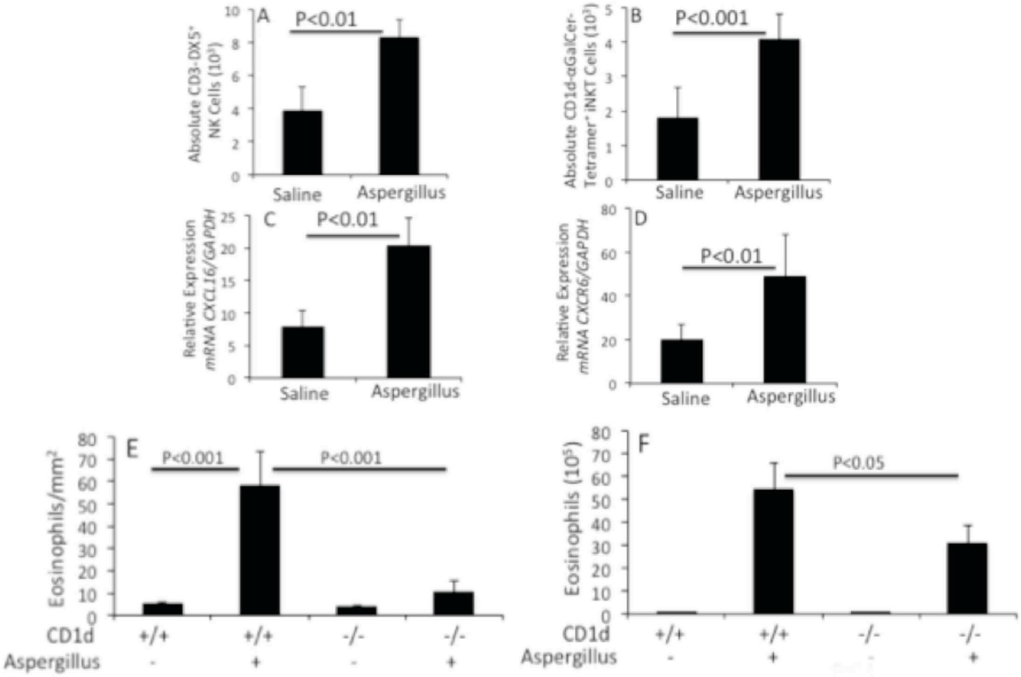

Supplementary figure 2. Induction of experimental EoE in NK cell-depleted mice. NK cells were depleted by 5 intravenous injections of anti-mouse asilo-GM1 antibody in mice (A, downward arrows). NK cell-depleted mice, IgG-injected control mice and untreated mice were exposed to intranasal allergen (*Aspergillus*) or saline following the experimental EoE protocol shown (A). The eosinophils induction in lung (BALF) and esophageal was determined. Levels of eosinophil in the BALF (B) and esophagus (C) of untreated, IgG control and NK cell-depleted mice following saline and allergen challenge are shown. Mice receiving allergen, IgG or anti-asiolGM1 antibody are indicated as (+) and those receiving saline as (-). Data is obtained 18-20 hrs after the last treatment and is expressed as mean ± SD (n = 9 mice/group).

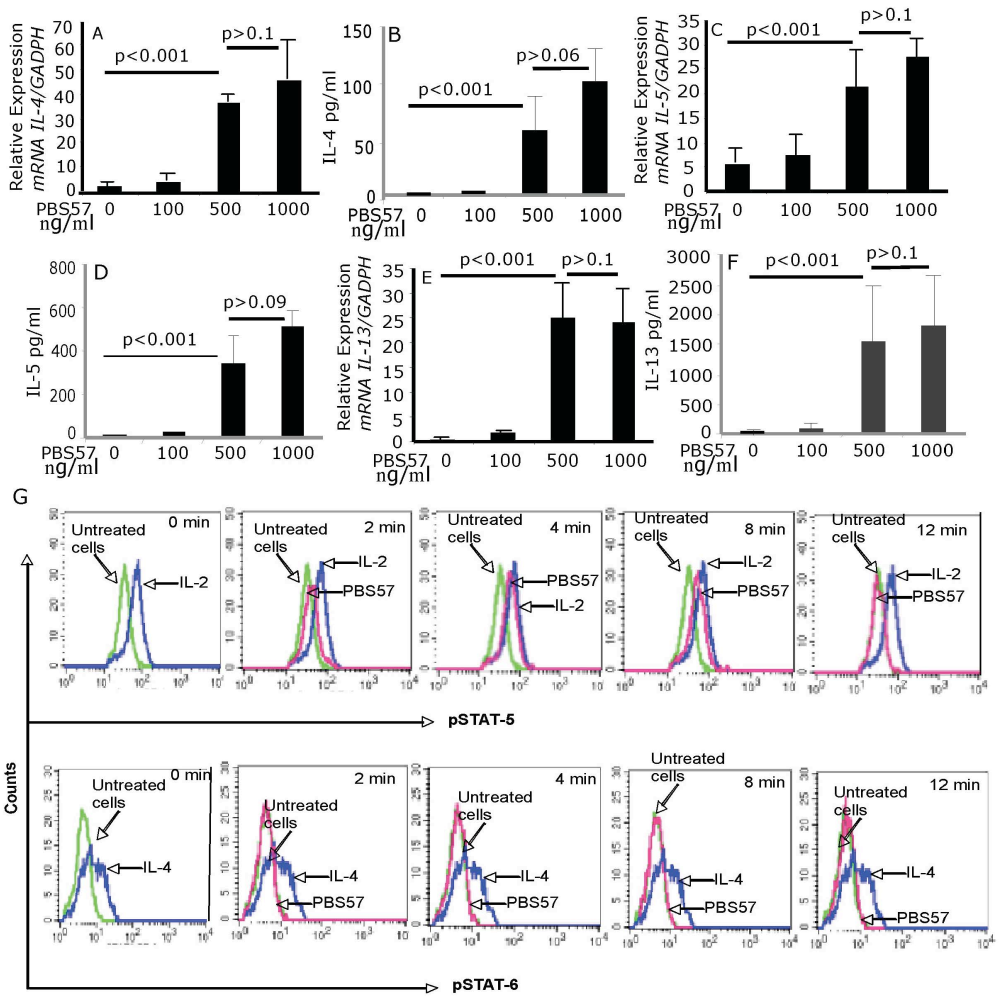

Supplimentary figure 3. iNKT cell and Th2 cytokine profiles following PBS57 exposure. A dose response (0, 100, 500 and 1000 ng/ml) analysis indicates that 24 hrs exposure of PBS57 activate iNKT cells and induces eosinophil active cytokines IL-4 mRNA and protein (A, B), IL-5 (C, D) and IL-13 (E, F). The cytokines mRNA expression was normalized with *β*-Actin and shown as a relative expression of each cytokine. Further, flow cytometric analysis was performed to examine *in vitro* activation of STAT family of molecules in PBS57 exposed iNKT cells. The PBS57 activated STAT5 phosphorylation is shown by flow cytometeric analysis, but no STAT6 activation

(pSTAT-6) by PBS57 was observed (A, B). In the histogram untreated cells were represented by green line, PBS57 treated with red line and blue line represent positive control (IL-2) treated for pSTAT5 and IL-4 treated for STAT6. Data is representative of three independent experiments.

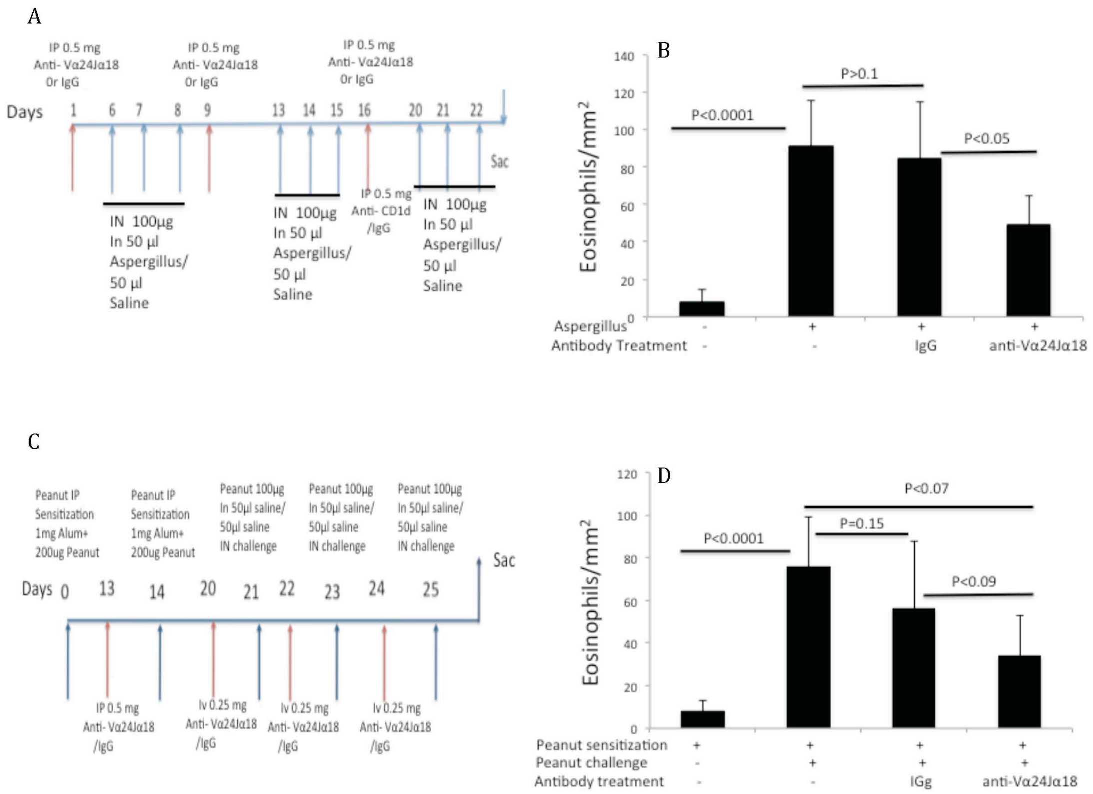

Supplementary figure 4. iNKT cell analysis in CD4 gene-deficient mice. iNKT cells were analyzed from total isolated cells from the spleen of CD4 gene-deficient mice using APC-conjugated CD1d-αGalCer-tetramer. The non-granulated total leukocyte

populations were gated (A) and analyzed for anti-CD3ε and anti-Vα24Jα18 and CD1d-αGalCer-tetramer. The anti-CD3ε and anti-Vα24Jα18 $^{+}$/CD1d-αGalCer-tetramer^{+} iNKT cells were detected in the cells isolated from the spleen of CD4 gene-deficient mice and a representative dot plot is shown (B), n = 3 mice
